# The influence of learning strategies and the environment on Chinese L3 English learners’ motivation to learn phonetic symbols: The mediating role of self-efficacy

**DOI:** 10.1371/journal.pone.0292398

**Published:** 2023-10-05

**Authors:** Jie Zeng, Yi Qin, Yu Zong, Tengfei Zhao

**Affiliations:** 1 School of Foreign Languages, Chengdu Normal University, Chengdu, Sichuan Province, China; 2 School of Education and Psychology, Chengdu Normal University, Chengdu, Sichuan Province, China; The University of Auckland, NEW ZEALAND

## Abstract

Previous studies have established a relationship between Chinese L3 English learners’ learning strategies, environment, self-efficacy, and motivation. However, limited research has examined these interconnections concerning Chinese L3 learners’ phonetic symbol learning (PSL), and it is hard to determine the extent or manner in which the aforementioned factors have an impact on the motivation toward PSL and their interactions among Chinese L3 English learners according to existing related studies. Structural equation modeling (SEM) can be utilized to tackle this question given its advantages in analyzing various factors in language learning motivation based on specific theories. This study, therefore, aims to investigate the direct and indirect effects of learning strategies and learning environment on the motivation towards PSL among Chinese L3 English learners and offer some pedagogical advice to teachers in Chinese L3 English instruction. To achieve this, a PSL Motivation Scale was developed, using data collected from 45 minority college students and analyzed via SEM. The results revealed that learning strategies and learning environment have direct impacts on the motivation towards PSL among Chinese L3 English learners, as well as indirect impacts on motivation through the mediation of self-efficacy. This study may provide a methodological and pedagogical reference for English pronunciation teaching in China and other contexts that we can stimulate students’ motivation toward PSL either directly or through the mediating effect of self-efficacy.

## 1. Introduction

According to Gardner et al. [1], one of the factors that affected proficiency in second language acquisition was motivation. Studies have shown that learning strategies have a considerable impact on motivation for L2/L3 learning [e.g., 2–5]. Furthermore, the influence of the learning environment on students’ learning was suggested by theories of ecology, socio-culture, and activity as well as the S-O-R (Stimulus-Organism-Response) model. Studies have shown that students’ motivation for language acquisition is substantially impacted by their sense of self-efficacy [6–8]. And the purpose of this study is to investigate how these variables relate to PSL for Chinese L3 English learners.

English has gained global prominence as an international language, spoken by both native English speakers and non-native English learners worldwide [9,10]. Graddol [11] categorized English speakers into three groups: (1) those who speak English as their first language (L1, approximately 375 million speakers); (2) those who speak English as a second or additional language (L2, also around 375 million speakers); and (3) those who learn English as a foreign language (EFL, approximately 750 million learners). Additionally, there is a substantial population of English speakers who use English as a third language (L3), such as ethnic minority English learners in China. With its diverse and sizable ethnic population, China is one of the most multilingual countries in the world [12]. As English education becomes more prevalent in elementary schools, an increasing number of minority students find themselves learning three languages simultaneously. This phenomenon is evinced in the following quote: "Most ethnic minority students learn their mother tongue, Chinese, and English simultaneously from an early age" [12].

However, studies indicate that minority students often lack motivation and clear goals in English learning, leading to pronunciation difficulties resulting from their unwillingness to learn phonetic symbols in multilingual contexts [13–15]. Chinese college students, in particular, face difficulties in learning English pronunciation [16] due to their limited understanding and learning of English phonetic symbols [17–19]. Some researchers argue that Chinese students’ pronunciation problems stem from a lack of motivation for learning phonetic symbols [12,20]. Moreover, although the latest Curriculum Plan and Curriculum Standards for Compulsory Education in China (2022 Edition) (CMOE) requires English phonetic symbols to be taught beginning in Grades 7 to 9 (i.e., from the age of 13), Mei and Wang [21] discovered that most students only start learning phonetic symbols at the university level. Therefore, there is a growing interest in understanding the attitudes and motivation of Chinese L3 English learners toward learning the language [22,23]. Specifically, it is essential to enhance the knowledge of the factors influencing the motivation of Chinese L3 English learners toward phonetic symbol learning (PSL).

Motivation is widely recognized as a crucial factor in non-native English speakers’ language learning and is considered a significant predictor of their language learning achievements [24–32]. Researchers, such as Dörnyei, Gardner, and Lambert [33], have thus delved into understanding how motivation impacts L2 acquisition. In particular, Gardner et al.’s [1] work is widely acknowledged as a leading influence in the field of L2 learning motivation [34–36]. Dörnyei [37] also asserted that motivation is the primary driving force for initiating L2 acquisition and sustaining the lengthy and often challenging learning process. Notably, Gardner and Lambert’s [38] series of studies on language learning motivation since 1959 found that while language aptitude contributes significantly to individual variation in language learning achievement, motivational factors can outweigh the effects of aptitude [39]. Educators also recognize motivation as one of the most crucial factors in L2 learning settings [40].

**Fig 1:**
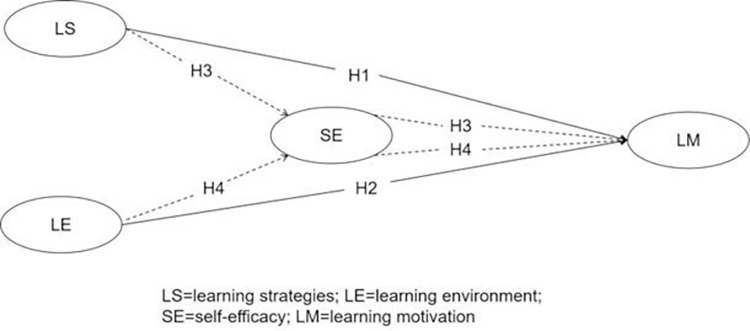
The research model for L3 English learners’ PSL.

Substantial research has proven that learning strategies, learning environments, and self-efficacy can influence the motivation of L2, L3, and EFL English learners [2,7,40–48]. Learning strategies and the learning environment are considered external factors that affect English learners’ motivation, which is an aspect of their internal psychology [49]. Some researchers believe that self-efficacy plays a mediating role in the impact of external social factors on individuals’ internal psychology [50,51]. However, no studies, to our best knowledge, have examined how Chinese L3 English learners approach PSL or how learning strategies and the learning environment affect their motivation to do so through self-efficacy. Therefore, building upon previous research, this study aimed to investigate the effects of learning strategies and the learning environment on the motivation of Chinese L3 English learners towards PSL, with a focus on the mediating role of self-efficacy in these effects. To investigate the aforementioned relationships, a research model (see Fig 1) and four corresponding hypotheses were developed and tested using structural equation modeling (SEM), which is a novel approach informed by relevant previous studies. The study was specifically conducted in the Chinese L3 context, providing valuable insights into the factors influencing students’ motivation toward PSL. The findings of this study contribute to the existing literature and serve as a foundation for future research on students’ PSL motivation in diverse contexts.

## 2. Related literature

### 2.1 The relationship between learning strategies and motivation

The concept of learning strategies in second language acquisition was first introduced in 1975 [52–54] and has been defined by various theorists [53]. One of the most commonly used definitions of learning strategies, provided by Oxford [55], is "steps or actions taken by language learners to enhance any aspect of their learning: acquisition, storage, retrieval, and use of information." Numerous studies examining the relationship between motivation and language learning strategies have found a significant correlation [56]. For instance, Wenden [57] demonstrated through case studies of two ESL students that the knowledge and use of strategies enhance students’ motivation, autonomy, and a sense of self-efficacy, as they feel better equipped to deal with challenges. It has also been observed that the utilization of language learning strategies is influenced by motivation, with highly motivated students employing strategies more frequently than their less motivated counterparts in various foreign language learning programs [58–61].

Similarly, Khodadad and Kaur [2] reported a significant positive correlation between learning strategies and motivation among Iranian EFL learners. Studies have also demonstrated the significant effect of learning strategies on motivation toward L3 learning [3–5,62–64]. However, limited research exists on the connection between learning strategies and the motivation of Chinese L3 English learners, specifically regarding PSL. Hence, we proposed the following hypothesis:

**H1.** Learning strategies directly influence Chinese L3 English learners’ motivation toward PSL.

### 2.2 The correlation between learning environment and motivation

Research on the educational environment traditionally focuses on individual knowledge, attitudes, and behavior [65]. The impact of the learning environment on students’ learning is supported by several theories, including theories of ecology, socio-culture, and activity. Ecology has been used as a metaphor in many theories of second language acquisition and socialization, promoting socio-cultural and socio-cognitive approaches to language acquisition research as well as socio-ethnographic approaches to studying socialization [66]. Human ecology theory focuses on how individuals interact with their environment as both biological and social beings [67], while language ecology, as defined by Haugen [68], refers to the study of interactions between a particular language and its environment.

Socio-cultural theory (SCT) is a psychological theory that emphasizes the important role of society in individual development. Drawing from Vygotsky’s ideas, SCT suggests that human cognition and higher mental functions develop over time, with social interactions serving as the starting point for cognitive and higher mental functions. Through engaging in social activities that require cognitive and communicative skills, individuals are drawn to utilizing these functions [69]. According to SCT, learning is primarily a social process; consequently, L2 learning research based on SCT investigates how language, cognition, and culture are acquired through the dynamic link between interaction and acquisition [70]. In other words, SCT views learning and mental development as processes that occur through the potential interaction between individuals and their environment [70]. Behroozizad, Nambiar [71] proposed the adoption of SCT to comprehend the acquisition of English as a foreign language, arguing that L2 learners receive interaction-based training to support their construction of social knowledge. They found that in an interaction-oriented classroom, the teacher’s scaffolding of learners’ ‘zone of proximal development’ mediates the students’ learning activities [71].

Activity theory, rooted in Soviet psychology with cultural-historical origins, emphasizes the concept of activity as an important aspect of human conduct and its connection to consciousness [72,73]. According to Rubinshtein [74], activity encompasses not only outward behavior but also its intimate connection with consciousness, highlighting the importance of understanding the interplay between consciousness and the natural world. Conscious learning, therefore, emerges from action or performance [73,75]. Kim [76] examined the motivational trajectories of Korean ESL learners in their L2 acquisition from an activity theory perspective, aiming to develop a non-reductionistic and genetic L2 motivation theory based on individuals’ unique experiences.

Although previous research has demonstrated a significant correlation between L2 learners’ learning environment and motivation [77,78], limited research has been conducted on the influence of the learning environment on the motivation of L3 English learners, specifically concerning English PSL. Therefore, this study attempted to explore this connection by proposing the following hypothesis:

**H2.** The learning environment directly influences Chinese L3 English learners’ motivation toward PSL.

### 2.3 The correlation between self-efficacy and motivation

Self-efficacy refers to an individual’s level of self-confidence in their ability to make decisions and produce desired outcomes [79]. It is closely related to an individual’s self-coping efforts [80]. As a person’s belief in their ability to perform a task [81], self-efficacy can predict performance even better than actual abilities or aptitude [82]. Self-efficacy also determines various aspects of task engagement, including task selection, effort, persistence, and the emotions associated with the task [83].

Schunk [84] discussed the potential role of self-efficacy in academic learning and highlighted its significance in predicting students’ performance in educational settings. He found that initial self-efficacy levels vary based on aptitude and prior knowledge, and students’ performance during tasks is influenced by situational and personal factors [41] such as goal setting and information processing. Students derive learning cues from these factors, which they use to assess their efficacy for further learning. When students believe they are making progress, their motivation increases, and they continue to have confidence in their ability to perform well as they complete assignments and develop their skills [85]. Likewise, when students believe in their ability to succeed, they are more motivated to perform well and remain engaged with a task, which is vital for academic success [82].

Studies have demonstrated that self-efficacy significantly impacts students’ motivation for language learning [6–8], including L2 learning [86]. Ersanlı [87] concluded that individuals with higher levels of academic self-efficacy and motivation exert more effort in learning a foreign language and are less likely to give up when facing challenges in the classroom setting. Overall, evidence suggests that self-efficacy has a positive influence on motivation in L2 learning, indicating that a stronger sense of self-efficacy often leads to better learning outcomes. However, few studies have examined how self-efficacy motivates L3 learning, highlighting the need for further research in this area.

### 2.4 Mediating role of self-efficacy

In the field of learning strategies, cognitive, behavioral, motivational, and emotional factors are often involved, which implies that learning strategies can influence individuals’ learning behaviors through the enhancement of their psychological and emotional states [88,89]. Self-efficacy refers to an individual’s belief in their ability to accomplish specific behavioral tasks [81]. It is a psychological state of the individual, and therefore, learning strategies may have an impact on self-efficacy, which in turn affects learning behavior. Previous studies have established that learning strategies have been found to positively predict self-efficacy [90,91]. Researchers have also discovered that self-efficacy significantly predicts learning motivation [92,93]. When individuals have a higher estimation of their learning abilities, it can stimulate stronger internal forces and result in a higher level of learning motivation. In conclusion, in the process of learning strategy implementation, self-efficacy may play an indirect role and collectively contribute to influencing individuals’ psychological and emotional states.

As explained earlier, numerous studies have shown a correlation between learning strategies and the motivation of L2 English learners. For example, Wong [94] found a significant positive correlation between the learning strategies and language self-efficacy of L2 graduate pre-service teachers, while Nosratinia et al.’s [95] study of 150 EFL students majoring in the English language revealed that self-efficacy predicts the use of EFL students’ language learning strategies.

The S-O-R (Stimulus-Organism-Response) model is one of the important theoretical models in modern cognitive psychology, which can be used to explain the influence of the environment on human behavior. The learning environment, as an objective stimulus (S), when individuals are in a good working or studying environment, favorable environmental conditions can enhance their psychological and physiological organism (O) like self-efficacy, ultimately affecting their specific response (R) in learning behavior. In previous studies, researchers have found that the work environment positively predicts self-efficacy [96,97]. Additionally, researchers have also discovered that self-efficacy plays a positive role in students’ learning motivation and engagement, as individuals with high self-efficacy demonstrate greater enthusiasm in their learning behaviors. When individuals are in a conducive learning atmosphere and superior learning environment, they are more likely to be influenced by the environment, stimulating their learning self-efficacy and exhibiting a more positive attitude towards learning tasks. Therefore, we propose H2: Self-efficacy plays a mediating role in the relationship between the learning environment and learning motivation.

This paper has established that learning strategies, learning environment, and self-efficacy are closely related to the motivation of L2 English learners. On the other hand, studies conducted in China have explored the connection between L3 English learning and self-efficacy [e.g., 98–100]. Among these, Yu [100] investigated Chinese L3 English learners in Guizhou and found that their self-efficacy levels are lower than those of L2 English learners. Similarly, researchers have explored the relationship between learning strategies and L2 self-efficacy [e.g., 42,101]. [42] found a strong and positive correlation between self-efficacy and the use of learning strategies. Gahungu [42] noted that learning strategies and self-efficacy are concepts that teachers and language professionals are increasingly familiar with, and varying degrees of attention have been given to these two constructs in second language learning research. Individuals with higher self-efficacy tend to outperform those with lower self-efficacy, such that students who employ language learning strategies are believed to be more autonomous, self-regulated, and capable of achieving a higher level of language proficiency [42]. This association between learning strategies and self-efficacy suggests that the former may indirectly predict the motivation of L3 learners through self-efficacy.

Meanwhile, though not directly related to learning English, some studies have demonstrated the importance of the learning environment [e.g., 102,103]. Notably, previous research has shown that self-efficacy mediates the relationship between the learning environment and EFL learning outcomes [104]. In particular, Han et al.’s [104] longitudinal study found the mediating role of self-efficacy in the correlation between Chinese university EFL learners’ perceptions of their online learning environment and learning outcomes. However, there is a lack of in-depth exploration of the relationship between learning strategies and self-efficacy in L3 learning and PSL. Therefore, we proposed the following hypotheses:

**H3.** Self-efficacy mediates the association between learning strategy and Chinese L3 English learners’ motivation towards PSL.

**H4.** Self-efficacy mediates the association between the learning environment and Chinese L3 English learners’ motivation towards PSL.

## 3. Methods

### 3.1 Participants

This study recruited adult college students from various majors at a public university in South-Western China beginning on 10 October 2022 and concluding on 10 December 2022. The number of participants in each phase of the entire experiment are stated in the following sections. Participants with different English language proficiency levels included those who learn English as L2 and L3. Among them, the students who learn English as L2 are Han students, and the L3 English learners are ethnic minority students. Participants were invited to complete anonymous questionnaires based on their preferences, and they were informed that they could withdraw from the study at any time. No personally identifiable information was shared before, during, or after this study. Before deciding to participate in this investigation, all participants gave their written informed consent. All participants gave their approval for publication. The research was conducted in accordance with the Helsinki Declaration and the American Psychological Association (APA) code of ethics. The Academic Ethics Committee of the School of Foreign Languages at Chengdu Normal University approved the study (SFLRA-2022003).

### 3.2 Pilot test

30 undergraduates majoring in English were invited to conduct a pilot test to evaluate the clarity, content wording, and expression of the initial scale questions adapted from different sources. We made adjustments to the scale accordingly. There are 31 items in the adjusted scale. To ensure the content validity of the scale, three English teachers and experts in the field of psychometry (including one professor with a doctorate degree) were invited to review the items and make modifications accordingly. The experts agreed that the theoretical composition of the scale was reasonable and the writing of the questions was consistent with the theory, and the initial scale of College Students’ Motivation toward PSL was preliminarily constructed.

### 3.3 Phase I. Development of a motivation scale for Chinese L3 English learners’ PSL

#### 3.3.1 Open-ended questionnaire survey and content analysis of responses

*3*.*3*.*1*.*1 Open-ended questionnaire survey*. An open-ended anonymous questionnaire was designed and distributed to a sample of 20 university students, with each student asked to provide a minimum of five responses to the question, "What motivates you to learn English phonetic symbols?" A total of 76 responses were collected from the participants.

*3*.*3*.*1*.*2 Content analysis of the responses*. In collaboration with two experienced English phonetics teachers and a psychology teacher, Bruner’s motivation theory [105] was combined with Gao’s classification of the English Learning Motivation Scale [106] to organize and categorize the 76 responses regarding PSL motivation. Entries that exhibited semantic repetition or unclear expressions were removed, resulting in the compilation of the English PSL Motivation Scale (Preliminary Version). This scale comprised 31 items and seven dimensions, namely intrinsic interest, learning situation, personal development, phonological awareness (communication medium), external requirements, English improvement, and performance improvement. The scale employed a five-point Likert scale, ranging from 1 (strongly agree) to 5 (strongly disagree), for measurement. All items were positively scored, and participants were instructed to respond truthfully based on their personal circumstances.

#### 3.3.2 Findings of exploratory factor analysis

Using the preliminary version of the English PSL Motivation Scale, we conducted a study at a university in the Sichuan Province of China. Convenience sampling was employed to select respondents from various majors and grades to answer the questionnaire, which was administered online. Out of the 340 questionnaires received, we excluded those that were duplicates from the same device, those with short response times, and those containing identical or habitual responses. This resulted in 323 valid questionnaires, consisting of 74 male and 249 female students.

To analyze the data, we performed exploratory factor analysis using SPSS. Initially, we conducted the Kaiser-Meyer-Olkin (KMO) test and Bartlett’s sphericity test. The KMO value was found to be 0.905, indicating that the sample was suitable for principal component analysis (p<0.001). Subsequently, factor analysis was conducted using principal component analysis and an orthogonal varimax rotation method. Based on the analysis, 21 items from the scale were retained according to specific criteria. Four factors emerged from the data, labeled as follows: learning English well; own interest; learning context; and external requirements. For further details, refer to the analysis results in Table 1.

**Table 1:** Exploratory factor analysis loadings matrix for the English PSL Motivation Scale (preliminary version), (N = 323).

Item	Factor 1	Factor 2	Factor 3	Factor 4
6. I learn phonetic symbols to master the foundation of English pronunciation.	0.728			
7. I learn phonetic symbols because I want to correct my pronunciation.	0.781			
11. I need to learn phonetic symbols well because it can improve my efficiency in communicating with others in English.	0.706			
13. I learn phonetic symbols so that I can learn English better.	0.768			
15. I learn phonetic symbols to facilitate independent learning of new words.	0.738			
20. I learn phonetic symbols to improve my sense of language.	0.668			
26. English is an important brick in the doorway of life, so it is important to learn phonetic symbols well.	0.774			
27. I learn phonetic symbols because they are the prerequisite and foundation for learning English well.	0.809			
28. I learn phonetic symbols so that I can spell words.	0.804			
29. I learn phonetic symbols in the hope that I can improve my listening or speaking performance.	0.755			
16. I think it is very interesting to learn phonetic symbols.		0.715		
4. I am learning phonetic symbols for teaching English when I become a teacher in the future.		0.527		
14. I have a special interest in learning phonetic symbols.		0.792		
17. I learn phonetic symbols because I need them for academic research.		0.652		
19. I learn phonetic symbols because I like the symbols themselves.		0.835		
8. My motivation to learn phonetic symbols depends a lot on whether I like my English class or not.			0.711	
5. My motivation to learn phonetic symbols depends a lot on whether I like my English teacher or not.			0.744	
9. I learn phonetic symbols because it is the learning content of the textbook itself.			0.729	
2. I started learning phonetic symbols because my teacher asked us to learn them.				0.634
30. I learn phonetic symbols because many people recommend learning them.				0.769
31. Many students who do well in English use phonetic symbols to mark words, and I learn phonetic symbols from them.				0.693

#### 3.3.3 Validation factor analysis

To further validate the preliminary questionnaire, we administered it once again to students from the same university, employing convenience sampling to gather participants for the online test. A total of 349 questionnaires were returned, and after eliminating invalid responses, we obtained 331 valid questionnaires. Among these, 79 participants were male and 252 were female.

Using MPLUS, we conducted a validation factor analysis on the collected data, the results of which are presented in Table 2. The findings indicate that most of the model’s indicators were favorable, with a comparative fit index (CFI) exceeding 0.9. Additionally, the model demonstrated a good fit, suggesting that the scale is suitable for measuring university students’ motivation toward PSL.

**Table 2:** Results of validated factor analysis of the PSL Motivation Scale (N = 331).

X^2^ /df	RMSEA	RMR	CFI	TLI
4.764	0.094	0.094	0.913	0.899

#### 3.3.4 Reliability test

The total scale exhibited good reliability, as evinced by an internal consistency reliability coefficient (α) of 0.88. This coefficient indicates that the items within the scale consistently measure the same construct, further validating the reliability of the scale.

#### 3.3.5 Determination of the scale

After carefully revising the items, we finalized the English PSL Motivation Scale (final version), which consists of four dimensions and 21 items. Reverse scoring was not used, and higher scores on the scale indicate a stronger motivation to learn English phonetic symbols. To assess the internal consistency of the questionnaire, we calculated Cronbach’s alpha coefficient, which was found to be 0.880. This coefficient indicates that the items within the scale are reliable and consistent in measuring the intended construct of motivation.

### 3.4 Phase II. Investigation of the relationships between learning strategies, environment, motivation, and self-efficacy in Chinese L3 English learners’ PSL

#### 3.4.1 Measurement scales

*3*.*4*.*1*.*1 Learning strategies scale*. The learning strategies scale used in this study was the Self-Identified Language Learning Strategies Scale (SILL) developed by Oxford [107]. This scale comprised 50 items and was rated on a five-point Likert scale from 1 (never do this) to 5 (always do this). The SILL included six subscales that measured learners’ metacognitive, cognitive, memory, compensatory, affective, and social strategies. Previous research has demonstrated high reliability for the SILL when translated into participants’ native language, with reported Cronbach’s alpha coefficients ranging from .91 to .94 for foreign language learners. In the current study, Cronbach’s alpha coefficient for the questionnaire was .969, indicating excellent internal consistency and reliability of the scale for measuring learning strategies in the context of Chinese L3 English learners.

*3*.*4*.*1*.*2 Learning environment scale*. The learning environment scale used in this study, developed by Ren [108], consisted of 41 items distributed across three dimensions: physical environment, human environment, and institutional environment. The scale demonstrated high reliability and validity. Participants responded to the questionnaire using a 5-point scale, ranging from strongly agree to strongly disagree. In this study, Cronbach’s alpha coefficient for the questionnaire was calculated as 0.959, indicating strong internal consistency.

*3*.*4*.*1*.*3 Self-efficacy scale*. The scale used in this study was adapted from the General Self-Efficacy Scale (GSES) developed by Schwarzer and Jerusalem [109]. It consisted of 10 items, each measured on a 4-point Likert scale ranging from 1 (completely incorrect) to 4 (completely correct). The response options were labeled as follows: 1 for “completely incorrect,” 2 for “incorrect,” 3 for “correct,” and 4 for “completely correct.” The scores obtained from the scale reflected the level of self-efficacy of the learners, with higher scores indicating greater self-efficacy. In this particular study, Cronbach’s alpha coefficient for the questionnaire was calculated as 0.917, indicating strong internal consistency.

*3*.*4*.*1*.*4 PSL motivation scale*. The self-developed English PSL Motivation Scale (final version) used in this study comprised 21 items and encompassed four dimensions assessing the motivation to learn phonetic symbols. Higher scores on the scale indicated a higher level of motivation. The questionnaire demonstrated good internal consistency reliability, with a Cronbach’s alpha coefficient of 0.880.

#### 3.4.2 Statistical process

As this study mainly focuses on Chinese L3 English learners’ motivation toward PSL, which is a special group of English learners in China. We followed Liu’s [110] sampling method and sample size requirements of the intermediate effect model, i.e., when α = 0.05, statistical testing power (1-β) is 0.80, and both a and b effect sizes are large, the minimum required sample size of the intermediate model test using the bootstrap interval method is 36 (p.287). The valid sample size for Chinese L3 English learners collected in this paper is 45, which meets the requirement. The 45 adult ethnic minority students (male n = 10, female n = 35) are from a university in Sichuan Province of China who study English as L3. These minority students were from different majors and grades with different English language proficiency and they learned Mandarin as L2 and English as L3, and none of them had lived outside Sichuan for more than three months. The PROCESS plug-in in SPSS [111] was used to analyze the data. The analysis involved correlation analysis, multiple regression analysis, and mediation analysis to explore the relationships and potential effects within the data.

## 4. Results

### 4.1 Descriptive statistics and correlation analysis

Descriptive statistics and correlation analysis were performed using the total scores of each variable and their dimension scores. The findings revealed significant correlations between motivation for PSL and learning strategies, learning environment, and self-efficacy for PSL. These correlations were observed in two distinct ways. A detailed summary of the results can be found in Table 3.

**Table 3:** Correlation coefficients between the variables (n = 45).

	Learning strategies	Learning environment	Self-efficacy in PSL	Motivation for PSL
Learning strategies	1			
Learning environment	.432**	1		
Self-efficacy in PSL	.463**	.445**	1	
Motivation for PSL	.562**	.526**	.443**	1

Note.

**means p < .01

*means p < .05, same below.

### 4.2 The structural model

The PROCESS plug-in in SPSS developed by Hayes and Scharkow [111] was employed to examine the direct and indirect effects of learning strategies, learning environment, and self-efficacy on the motivation to learn English phonetic symbols. In the regression models, learning strategy and learning environment were treated as the predictor variables, motivation for PSL was the outcome variable, and self-efficacy in PSL was the mediating variable.

Table 4 presents the regression analysis results. When both learning strategies and self-efficacy were included in the regression equation, they were found to significantly and positively predict motivation (learning strategies: β = 0.4549, p<0.001 and self-efficacy: β = 0.2324, p<0.005). These findings support Hypothesis 1 and also suggest that self-efficacy may act as a mediator between learning strategies and motivation for PSL. Likewise, when learning environment and self-efficacy were simultaneously entered into the regression equation, the learning environment positively predicted motivation (β = 0.411, p<0.001), as did self-efficacy (β = 0.260, p<0.01). Furthermore, the learning environment was found to have a positive influence on both motivation (β = 0.526, p<0.001) and self-efficacy (β = 0.445, p<0.01). These results validate Hypothesis 2 and suggest that self-efficacy may mediate the relationship between the learning environment and motivation for PSL.

**Table 4:** The effects of learning strategy, learning environment, and self-efficacy on motivation towards PSL (n = 45).

		Standardized regression coefficients and significance tests				Equation interpretation capability		
Result Variables	Predictive variables		β	t	95% confidence interval	R	R^2^	F
Motivation for PSL	Learning Strategies		0.4549	3.2629***	[0.2482,0.3522]	0.599	0.358	11.748***
	Self-efficacy		0.2324	1.6669**	[0.3090,0.4129]			
Self-efficacy in PSL	Learning Strategies		0.4629	3.4247**	[0.3257,0.4322]	0.463	0.214	11.728**
Motivation for PSL	Learning Strategies		0.5624	4.4603***	[0.1696,0.7196]	0.562	0.316	19.895***
Motivation for PSL	Learning Environment		0.411	2.915***	[0.2482,0.3522]	0.576	0.331	10.748***
	Self-efficacy		0.260	1.846**	[0.3090,0.4129]			
Self-efficacy in PSL	Learning Environment		0.445	3.260**	[0.048,0.204]	0.445	0.198	10.728**
Motivation for PSL	Learning Environment		0.526	4.061***	[0.3852, 4888]	0.526	0.316	19.895***

The regression analysis with learning strategies, and self-efficacy as predictors revealed that the overall regression equation was significant (R^2^ = 0.562, p<0.001), indicating that learning strategies, self-efficacy in PSL, and motivation for PSL were significantly correlated. To assess the mediating effect, bootstrap sampling was performed. The results showed that the indirect effect of the path with self-efficacy as the mediating variable was significant, confirming Hypothesis 3. The mediating role of self-efficacy in learning strategies’ impact on motivation for PSL was thus supported. Further details can be found in Table 5.

**Table 5:** Results of the indirect effect of learning strategies on motivation for PSL via self-efficacy (n = 45).

	Effect Value	Boot SE	95% confidence interval	Effectiveness share (%)
Total effect (c)	0.562	0.0264	[0.315, 0.810]	
Direct effect (c’)	0.463	0.0265	[0.182, 0.728].	82.38
Indirect effects (a*b)	0.108	0.0159	[-0.003 ~, 0.318]	17.62

The results of the regression model which included learning environment, self-efficacy, and motivation for PSL indicated that the overall regression equation was significant (R^2^ = 0.240, p<0.001), suggesting significant relationships between the three variables. To assess the mediating effect, bootstrapping was utilized. The results demonstrated that the indirect effect of the path from the learning environment to motivation with self-efficacy as the mediating variable was significant, confirming Hypothesis 4. This finding provides evidence for the mediating effect of self-efficacy in the effect of the learning environment on motivation towards PSL. Further information can be found in Table 6.

**Table 6:** Results of the indirect effects of learning environment on motivation for PSL via self-efficacy (n = 45).

	Effect Value	Boot SE	95% confidence interval	Effectiveness share (%)
Total effect (c)	0.240	0.059	[0.121, 0.359]	
Direct effect (c’)	0.187	0.064	[0.058,0.317].	77.92
Indirect effects (a*b)	0.053	0.032	[-0.003, 0.123]	22.08

Testing the significance of a

*b is used to determine whether there is a mediating effect or not, a practice known as the product coefficient test. In this study, a (p<0.001) and b (p<0.001) are both significant, and the indirect effect of ab and direct effect c’ are significant, and both of the two effects are positive. Therefore, we believe that there is a partial mediating effect. Thereafter, we also examined the influence of indirect effects on the total effect as an explanation for the influence of self-efficacy in the model (see Table 6).

**Fig 2:**
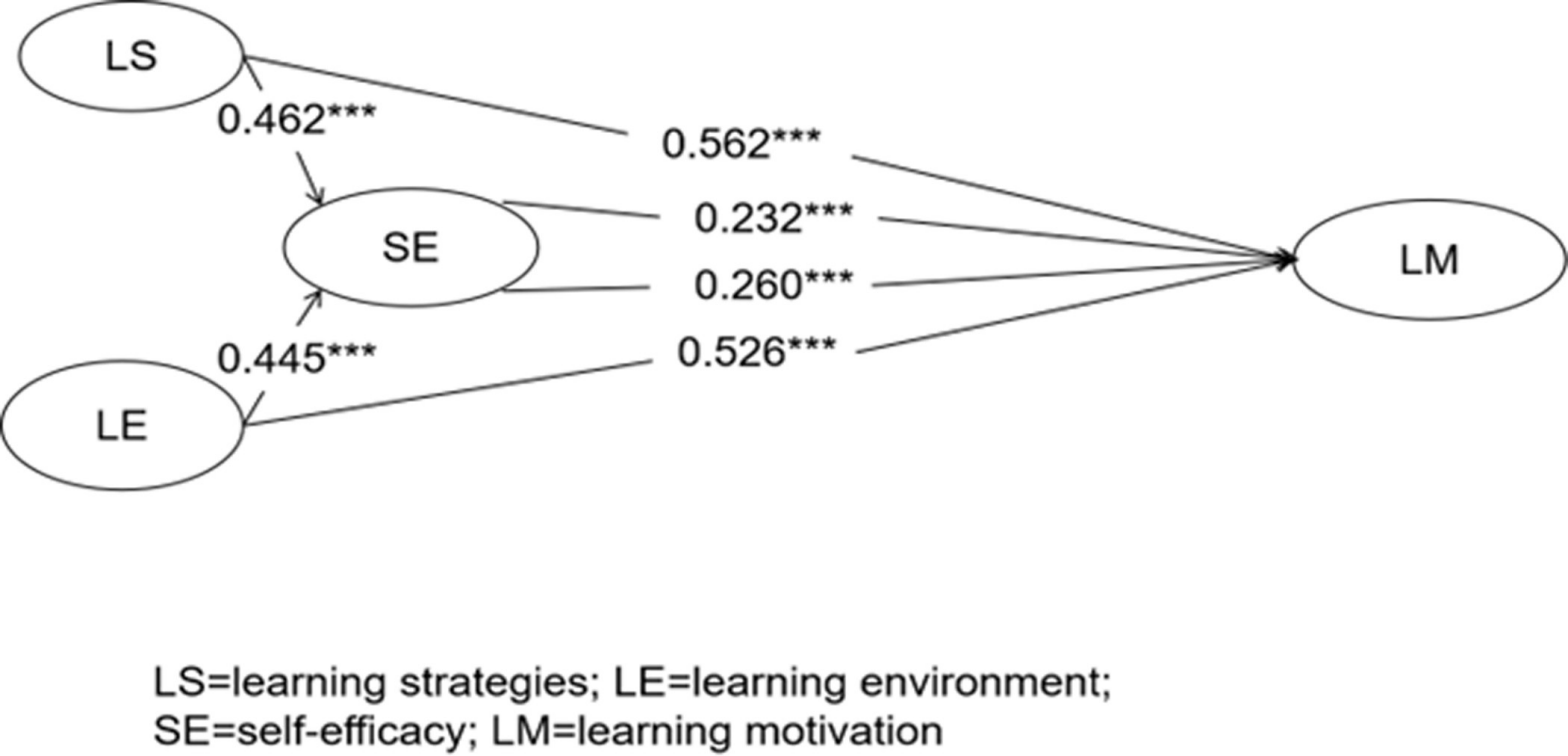
The results of structural model testing.

Based on the results discussed above, all four hypotheses put forth in this study were confirmed, and the PSL model of Chinese L3 English learners was validated. The findings indicate that learning strategies and learning environment have a direct impact on the PSL motivation of L3 English learners. Additionally, these factors indirectly influence PSL motivation through the mediating role of self-efficacy. The results of the hypotheses can be found in Table 7, while the structural equation model testing findings are illustrated in Fig 2.

**Table 7:** Hypotheses testing results.

Hypothesis	Relationship	Std. Beta	*t*-value	*p*-value	Decision	VIF
H1	LS>LM	0.562	4.460	<0.001	supported	1.981
H2	SE>LM	0.232	1.667	<0.001	supported	1.987
H3	LE>LM	0.526	4.061	<0.001	supported	1.987
H4	SE>LM	0.260	1.846	<0.001	supported	1.987

Note. LS = learning strategies; LM = learning motivation; SE = self-efficacy; LE = learning environment.

## 5. Discussion

The impacts of learning strategies, the learning environment, and self-efficacy on the motivation of Chinese L3 English learners toward PSL were examined in this study. To address the research questions, this section discusses the research findings.

### 5.1 The relationship between learning strategies and motivation

The first research question addressed whether learning strategy usage directly predicts Chinese L3 English learners’ motivation for PSL. The study’s results indicate a significant positive correlation between learning strategies and these learners’ motivation for PSL (r = 0.56, p<0.01). This finding is consistent with previous studies that have demonstrated the significant positive effect of learning strategies on the motivation of Chinese L2 English learners [e.g., 112,113]. Nonetheless, while previous studies primarily focused on the relationship between vocabulary learning strategies and motivation [e.g., 113–115], this paper investigated the motivation of Chinese L3 English learners in acquiring English phonetic symbols. Regarding Chinese L3 English learners’ motivation towards English PSL, we divided motivation into four dimensions: learning English well, own interest, learning context, and external requirements. Among these dimensions, the strongest positive correlation was found between learning strategy use and own interest (r = 0.59, p<0.01), which aligns with the findings of Shen and Chen [116]. However, we did not find a significant positive association between learning strategy use and learning context.

### 5.2 The correlation between learning environment and motivation

The second research question aimed to investigate whether the learning environment directly predicts Chinese L3 English learners’ motivation for PSL. Our study demonstrates a significant positive relationship between the learning environment and Chinese L3 English learners’ motivation toward PSL (r = 0.52, p<0.01). This finding corroborates previous research conducted by Ekiz and Kulmetov [40] and Wu [77], which revealed that a better learning environment leads to higher motivation among L2 English learners. Additionally, the results highlight that the most significant positive relationship is between the learning environment and external requirements (r = 0.50, p<0.01). As argued by Wei [117], both the learning environment and external requirements are crucial factors influencing students’ learning motivation. However, we did not observe a significant positive association between the learning environment and the learning context. According to many researchers who have discussed prior studies, the learning context and learning environment are closely related. Nevertheless, this study’s definition of learning context—which is different from the learning environment of material circumstances since it relates to the influence of learning atmosphere brought by others—is at odds with the findings of earlier studies. The aforementioned outcomes which differ from previous studies might be due to this.

### 5.3 The mediating role of self-efficacy

The third research question aimed to investigate whether learning strategies influence Chinese L3 English learners’ motivation for PSL through self-efficacy. The study initially examined the relationship between learning strategy use and self-efficacy, revealing a positive correlation between the two. In other words, students who employ learning strategies are more likely to feel more confident in their ability to learn, which is consistent with previous studies [42,101]. This study found that self-efficacy mediates the impact of learning strategies on motivation toward PSL. This finding is consistent with the work of Akamatsu, Nakaya [118], who investigated the mediating role of self-efficacy in the relationship between metacognitive strategies and self-regulating learning processes.

The fourth research question examined whether the learning environment influences Chinese L3 English learners’ motivation to learn English phonetic symbols through self-efficacy. The study indicates a positive correlation between the learning environment and self-efficacy, in line with Lorsbach and Jinks’s [119] argument that self-efficacy is influenced by elements of the learning environment, including expectations, goals, and rewards. We further found that the learning environment can predict Chinese L3 English learners’ motivation for PSL through self-efficacy, supporting its mediating role. This conclusion corresponds with the findings of Fast, Lewis [120], who revealed a minor but significant mediating effect of self-efficacy between classroom atmosphere and math performance. Additionally, the mediating influence of self-efficacy observed in this research aligns with the findings of Li and Yin [50], who highlighted that self-concept mediates the influence of external social factors on individuals’ internal psychology. Looking at earlier research, we did not discover that the SEM approach was utilized to discuss the study of Chinese L3 learners’ motivation for English PSL; hence, this study fills this gap.

## 6. Conclusion

The study utilized a structural equation model to examine the interactions among learning strategies, the learning environment, self-efficacy, and Chinese L3 English learners’ motivation towards PSL, aiming at addressing a theoretical gap in the field. It provides insights into the connections between these variables in the Chinese L3 context, serving as a valuable research tool for future investigations in various settings. This study may provide a theoretical and applied reference for English pronunciation teaching in China and other contexts. On the one hand, it is necessary to directly stimulate students’ motivation in PSL. In addition, this study found the mediating effect of self-efficacy, thus providing a new way and method to stimulate students’ motivation toward PSL through this mediating effect.

The findings of this study contribute to a better understanding of the factors influencing Chinese L3 English learners’ motivation for PSL. By considering learning strategies, the learning environment, self-efficacy, and motivation within a structural model, the study sheds light on how each factor impacts L3 motivation. These results have implications for teachers involved in Chinese L3 English instruction. First, the importance of learning strategies and the learning environment for Chinese L3 English learners’ motivation toward PSL is underscored in this study. Consequently, we encourage teachers to promote the use of effective learning strategies among students. They should also strive to develop appropriate learning strategies and enhance students’ ability to engage in independent learning.

Furthermore, teachers can enhance students’ learning effectiveness and self-efficacy by implementing suitable learning techniques in the autonomous learning process. By teaching students how to apply tactics and helping them select the most suitable ones, teachers can simplify the learning process and empower students to take ownership of their learning. Additionally, workshops on active learning strategies and exposure to various language learning methodologies can boost students’ motivation for PSL. Teachers can also emphasize student involvement in language learning, provide real-world practice opportunities, and strive to create a positive, supportive, friendly, peaceful, relaxed, and enjoyable classroom environment that facilitates language learning and PSL. To provide students with a conducive linguistic context for learning English phonetic symbols, teachers should carefully plan classroom instruction and prioritize small group work as well. Meanwhile, students should be given more choices and opportunities to express their opinions. By implementing these strategies, teachers can better support students in their language learning journey.

The results of the study highlight the strong connection between learning strategies and self-interest as motivation in Chinese L3 English learners’ PSL. Additionally, the study indicates a significant relationship between the learning environment and external requirements. It is important to consider these findings. Interest is recognized as a content-specific motivational feature, comprising inherent feeling-related and value-related aspects [121]. Early modern psychology pioneer Herbart [122] emphasized the importance of interest in education and believed that learning and interest are closely intertwined. Developing students’ broad and multifaceted interests is crucial to fostering meaningful learning, long-term knowledge retention, and the motivation to pursue further study. Therefore, teachers should strive to spark students’ interest by employing creative and exciting teaching methods. As English phonetic symbols often receive low student interest, assignments and activities that challenge learners to increase their attention and engagement with phonetic symbols are necessary, while promoting the use of effective learning strategies.

External requirements have been identified as a contributing factor to reduced student motivation. Wei’s [123] research on Chinese undergraduates majoring in non-English subjects supports this notion. Teachers should thus guide students on how to effectively handle external requirements. Encouraging students to set their learning objectives and choose content, particularly when their intrinsic motivation is low, can be beneficial. Counseling and guidance on career and life planning can also help alleviate psychological tension and anxiety caused by external requirements. It is essential to develop explicit training programs and learning tasks to enhance Chinese L3 English learners’ motivation for PSL. Additionally, addressing the lack of qualitative improvement in the learning environment requires prioritizing the promotion of external requirements.

The study findings on self-efficacy also have implications for English teachers and learners in China. The research underscores the significant impact of self-efficacy on the motivation of Chinese L3 English learners. Since Chinese L3 English learners need to address the issue of poor motivation caused by their weak English foundation, teachers should focus on boosting students’ confidence, addressing their psychological needs, and inspiring their motivation to learn English phonetic symbols. Timely support and assistance from teachers are crucial for the successful acquisition of English by Chinese L3 learners. It is important to convey to students that learning English phonetic symbols is not an insurmountable task.

Despite these implications, this study has several limitations. The sample size was relatively small, consisting of participants from a single university in one province of China, and with lower-intermediate English language proficiency. We suggest that future studies strive for more diverse samples, encompassing varying language proficiency levels and linguistic contexts. Additionally, there was an imbalance in gender representation, with significantly more female participants. Further research is needed to explore the potential interactions between variables concerning gender differences.

## Supporting information

S1 File English phonetic symbols learning questionnaires. (PDF)
