# Supplementary material for: The influence of learning strategies and the environment on Chinese L3 English learners’ motivation to learn phonetic symbols: The mediating role of self-efficacy
Source: PLoS One. 2023 Oct 5;18(10):e0292398. doi: 10.1371/journal.pone.0292398 (PMC10553281; doi:10.1371/journal.pone.0292398)
Supplement: S1 File — (PDF) [file pone.0292398.s001.pdf]

Appendix A

English phonetic symbols learning questionnaires

(This is the English version of the Chinese questionnaires that we accuturally used in our study. The Chinese questionnaires are also at the end of this document.)

Hello, dear students! The purpose of this questionnaire is to find out the difficulties and factors influencing the learning of phonetic symbols in college students. Please take a little time to read the questions carefully and then answer them according to your real situation. This questionnaire is anonymous, and the answers are not right or wrong, and will be used for research purposes only. You can withdraw from this study at any time.

Part I Basic Information

1. Gender: ① Female ②Male

2. Grade: ①Freshman year ②Sophomore year ③Junior year ④Senior year

3. Ethnicity: ① Han Chinese ②Yi ③Qiang ④Tibetan ⑤Other ________

4. Home location. ①Rural ②Township ③County city ④Urban ⑤Other ________

5. English score in the college entrance examination: ① above 130 ② 110-129 ③ 90-109 ④ 70-89 ⑤ below 69

6. tem-4 (English majors) or cet-4 (non-English majors) scores: ___________________

7. I think my level of mastery of phonetic symbols recognition: ① very good ② good ③ average ④ bad ⑤ very bad

I base this on ___________________________________

Part II Phonetic Symbols Learning Self-Efficacy Scale

The following 10 sentences are about how you usually think about your phonetic learning. Please answer according to your actual situation (actual feeling). There is no right or wrong answer, and you do not need to think much about each sentence. 1 means "completely incorrect", 2 means "somewhat correct", 3 means "mostly correct", 4 means "completely correct".

**Item**	**Not at all correct**	**Somewhat correct**	**Mostly correct**	**Completely correct**
1. If I try my best to learn	1	2	3	4

English phonetics, I can always solve the problem.				
2. I am capable of learning phonetic symbols even when others find it difficult.	1	2	3	4
3. For me, it was easy to stick to the ideal of learning phonetic symbols and reaching my phonetic learning goals.	1	2	3	4
4. I am confident that I can handle anything phonetic learning effectively.	1	2	3	4
5. With my talents, I can handle unexpected situations in phonetic learning.	1	2	3	4
6. If I put in the necessary effort, I will be able to solve most of my phonetic learning challenges.	1	2	3	4
7. I can face phonetic learning difficulties calmly because I trust my ability to deal with phonetic learning problems.	1	2	3	4
8. When faced with a phonetic learning problem, I can usually find several solutions.	1	2	3	4
9. When I have trouble learning phonetic symbols, I can usually think of some ways to cope with it.	1	2	3	4

10. I can handle whatever phonetic learning related things happen to me.	1	2	3	4

Part III Motivation Scale for Learning Phonetic symbols

The following questions are about some practices in the process of learning phonetic symbols, please choose the corresponding option according to your actual situation. 1 means "strongly disagree", 2 means "disagree", 3 means "general", 4 means "agree", 5 means "strongly agree".

**Item**	**Strongly agree**	**Agree**	**General**	**Disagree**	**Strongly disagree**
1. Many students with good English grades use phonetic symbols to mark words, and I learn phonetic symbols from them.	5	4	3	2	1
2. I learn phonetic symbols because they are the prerequisite and foundation for learning English well.	5	4	3	2	1
3. I learned phonetic symbols because many people recommended learning them.	5	4	3	2	1
4. I am learning phonetic symbols for teaching English when I become a teacher in the future.	5	4	3	2	1
5. My motivation to learn phonetic symbols depends a lot on whether I like my English teacher or not.	5	4	3	2	1
6. I learn phonetic symbols in order to master the foundation of	5	4	3	2	1

English pronunciation.					
7. I learned phonetic symbols because I wanted to correct my pronunciation.	5	4	3	2	1
8. My motivation to learn phonetic symbols depends a lot on whether I like my English class or not.	5	4	3	2	1
9. I learned the phonetic symbols because it is the learning content of the textbook itself.	5	4	3	2	1
10. I learn phonetic symbols in the hope that I can improve my listening or speaking performance.	5	4	3	2	1
11. It is important for me to learn phonetic symbols well because it can improve my efficiency in communicating with others in English.	5	4	3	2	1
12. Learning phonetic symbols well gives me a sense of accomplishment.	5	4	3	2	1
13. I learn phonetic symbols so that I can learn English better.	5	4	3	2	1
14. I have a special interest in learning phonetic symbols.	5	4	3	2	1
15. I learn phonetic symbols to facilitate independent learning of new words.	5	4	3	2	1
16. I think it is very interesting to learn phonetic symbols.	5	4	3	2	1
17. English is an important brick in the doorway of life, so it is	5	4	3	2	1

important to learn phonetic symbols well.					
18. I learned phonetic symbols so that I could spell words.	5	4	3	2	1
19. I learned phonetic symbols because I like the symbols themselves.	5	4	3	2	1
20. I learn phonetic symbols to improve my sense of language.	5	4	3	2	1
21 I learned phonetic symbols because my teacher emphasized the importance of phonetic symbols	5	4	3	2	1

Part IV Learning Strategies Scale

This questionnaire is designed to investigate your use of language learning strategies. 1 means "never do this", 2 means "hardly ever do this", 3 means "sometimes do this", 4 means "often do this",5 means "always do this". The number that matches your actual situation and circle it.

Item	Never	Almost not	Sometime	often	Completely true
1.When I learn English, I make connections between what I have learned and what I have learned, and build a network of relationships.	1	2	3	4	5
2.I use the newly learned words in sentences to deepen my memory.	1	2	3	4	5
3.I try to memorize words by combining the sound, form and meaning of the words.	1	2	3	4	5
4.I remember the word by associating it in my mind with the situation in which it applies.	1	2	3	4	5
5.I use the sound of the words to memorize the words (e.g., rice and ice).	1	2	3	4	5
6.I use vocabulary cards to recite new words.	1	2	3	4	5
7.I use body language to remember new words.	1	2	3	4	5
8.I often review words or texts that I have learned.	1	2	3	4	5
9.I remember a word by remembering where it appears on a page or in a	1	2	3	4	5

word list.					
10.I read or write new words many times over.	1	2	3	4	5
11.I imitate the way native English speakers speak.	1	2	3	4	5
12.I practice my English pronunciation.	1	2	3	4	5
13.I practice the English words I have already mastered in various ways.	1	2	3	4	5
14.I often speak English to myself or talk to my classmates and teachers in English.	1	2	3	4	5
15.I watch English programs, English movies or listen to English radio.	1	2	3	4	5
16.I read English as a pleasure.	1	2	3	4	5
17.I take notes, write letters, messages or reports in English.	1	2	3	4	5
18.When I read the article, I skim through it quickly and then re-read it carefully.	1	2	3	4	5
19.When I learn new words, I always try to find Chinese words that correspond to or are close to their meaning.	1	2	3	4	5
20.I try to summarize some fixed patterns in English such as verb collocation.	1	2	3	4	5
21.I break down new words (especially multi-syllabic ones) into small meaningful parts (e.g. pre, middle, suffix or cognate words, etc.) to understand the meaning of the whole word.	1	2	3	4	5
22.In reading, I try not to translate English into Chinese word by word.	1	2	3	4	5
23.I can summarize what I have heard or read about English.	1	2	3	4	5
24.For unfamiliar words, I guessed their meanings.	1	2	3	4	5
25.I use gestures when I can't think of a word to express my meaning in an English conversation.	1	2	3	4	5
26.When I don't know which word to use to express what I mean, I make up a word.	1	2	3	4	5
27.When I read English articles, I don't look up the meaning of every single word.	1	2	3	4	5
28.When having a conversation with someone in English, I guess what they are going to say next.	1	2	3	4	5

29.If I can't think of an appropriate word, I use a word or phrase with a similar meaning.	1	2	3	4	5
30.I try to use the new words I learn consciously in various English .	1	2	3	4	5
31.I will pay attention to the mistakes I make in using English, and correcting them will help me learn English better.	1	2	3	4	5
32.I always pay attention when people speak English.	1	2	3	4	5
33.I've been trying to figure out how to learn English effectively.	1	2	3	4	5
34.I plan enough time to study English every day or at every stage.	1	2	3	4	5
35.I always find someone to practice speaking with me.	1	2	3	4	5
36.I read as many English articles as I can.	1	2	3	4	5
37.I have clear goals for improving my English skills.	1	2	3	4	5
38.I often reflect on my English learning method, constantly summarize the strengths and weaknesses, and constantly improve.	1	2	3	4	5
39.Whenever I have a fear of using English, I try to relax my nerves.	1	2	3	4	5
40.Although I was afraid of making mistakes, I encouraged myself to speak English.	1	2	3	4	5
41.When I do well in English, I reward myself in my own way.	1	2	3	4	5
42.I watch for nervousness when I learn or use English.	1	2	3	4	5
43.I write down my feelings about learning English in my diary.	1	2	3	4	5
44.I share my experiences and feelings with others when learning English.	1	2	3	4	5
45.When I don't understand someone's English, I ask the speaker to speak slower or repeat it.	1	2	3	4	5

46.When I speak English, I ask the English speaker to correct my English expressions.	1	2	3	4	5
47.I practice English with other students.	1	2	3	4	5
48.I actively seek the help of native English speakers or English teachers.	1	2	3	4	5
49.I take the initiative to ask people questions in English.	1	2	3	4	5
50.I take the initiative to learn about the culture of English-speaking countries.	1	2	3	4	5

Part V Learning Environment Scale

1. If the content of each question is consistent with your current situation, please choose agree or strongly agree; if it is not consistent with your current situation, please choose disagree or strongly disagree. For questions where it is difficult to make a choice, please choose neutral. 4 means "very agree",3 means" agree",2 means" neutral",1 means "disagree",0 means "very disagree".
2. Thank you for your participation!

**Item**	**Strongly agree**	**Agree**	**Neutral**	**Disagree**	**Strongly disagree**
1.The school has a special place for practical English training.	4	3	2	1	0
2. Teachers in English classes often use multimedia equipment to teach.	4	3	2	1	0
3. The campus network provides ample web links to internal resources (e.g., past year English papers, lecture notes).	4	3	2	1	0
4. English class schedule is reasonable.	4	3	2	1	0
5. The campus network provides sufficient web links to external resources (e.g., English-related course websites).	4	3	2	1	0
6. The teacher has used relevant online tools (e.g.QQ,	4	3	2	1	0

blogs, BBS, etc.) to assign homework and answer questions to students.					
7. English has a corresponding practical course schedule.	4	3	2	1	0
8. Our English tests focus more on students' written skills and the majority of our English listening and speaking training is in the speech classroom.	4	3	2	1	0
9. My English teacher was not an authority, but a friend who was my equal.	4	3	2	1	0
10. We often use online English materials.	4	3	2	1	0
11. The English teacher is very knowledgeable and insightful.	4	3	2	1	0
12. I often study English with the help of extra-curricular materials (reference books, English periodicals and magazines, etc.).	4	3	2	1	0
13. I have taken other English courses.	4	3	2	1	0
14. This English textbook requires students to use a problem-solving approach to learning.	4	3	2	1	0
15. Teachers always assess our English level based on paper grades.	4	3	2	1	0
16. I have submitted assignments and	4	3	2	1	0

communicated with the instructor through relevant online tools (e.g., QQ, blog, BBS, etc.).					
17. English teachers focus on students' self-assessment of English learning.	4	3	2	1	0
18. The assignment topics at the end of the lesson are well designed.	4	3	2	1	0
19. My study place after school is mainly in the classroom.	4	3	2	1	0
20. The students in our class are highly motivated to learn English.	4	3	2	1	0
21. The teacher assigns different assignments according to the learning situation of the students.	4	3	2	1	0
22. I often work with my classmates to complete English learning tasks.	4	3	2	1	0
23. It is easy to find English materials in the study place.	4	3	2	1	0
24. Often organizations like the New Oriental School come to the school to provide English learning instruction.	4	3	2	1	0
25. English teachers have a high level of information technology (e.g., multimedia application skills, computer application skills).	4	3	2	1	0
26. The English teacher has a good rapport	4	3	2	1	0

with us.					
27. The current amount of English class time is just right.	4	3	2	1	0
28. English teachers often provide materials to encourage students to learn on their own.	4	3	2	1	0
29. I can always get help from my English teacher.	4	3	2	1	0
30. English classrooms are equipped with appropriate multimedia equipment.	4	3	2	1	0
31. I would recommend to others to use our teaching materials.	4	3	2	1	0
32. Feedback from English teachers is encouraging.	4	3	2	1	0
33. I can get responses from other students to the English questions I ask and organize role-playing exercises for students.	4	3	2	1	0
34.I can get responses from other students to the English questions I ask.	4	3	2	1	0
35. English classes are mainly taught by the teacher.	4	3	2	1	0
36. English teacher with standard pronunciation and fluent speaking.	4	3	2	1	0

37. My English queries will be responded to by the webmaster.	4	3	2	1	0
38. The school rewards students for excellence in English learning accordingly.	4	3	2	1	0
39. English teachers actively lead students to participate in discussions.	4	3	2	1	0
40. English textbooks provide a number of effective independent learning strategies (specific individual learning methods).	4	3	2	1	0
41. English teachers often set up situations and organize role-playing exercises for students.	4	3	2	1	0

英语音标学习问卷调查（中文实测版）

亲爱的同学，您好！这是一份关于大学生音标学习的调查问卷，目的是想了解大学生在音标学习过程中的困难与影响因素，您的回答将为我们的研究提供宝贵的依据，也为大学生音标的学习提供重要帮助。请花费一点宝贵时间仔细阅读题目后根据您的真实情况进行作答。本问卷不记名，答案也无对错之分，仅作研究使用，对您的回答我们也会绝对保密，您可以随时退出本研究。感谢您参与本次调查。

第一部分 基本信息

1.性别：①女 ②男

2.年级：①大一 ②大二 ③大三 ④大四

3.民族：①汉族 ②彝族 ③羌族 ④藏族 ⑤其他_______

4.家庭所在地： ①农村 ②乡镇 ③县城 ④市区 ⑤其他_______

5.高考英语分数：①130 以上 ②110-129 ③90-109 ④70-89 ⑤69 以下

6.tem-4（英语专业）或 cet-4（非英语专业）成绩：___________________

7.我认为自己的音标认读掌握水平：①非常好　②好　③一般　④不好　⑤非常不好

我的依据是______________________________________

第二部分 音标学习自我效能感量表

指导语：以下 10 个句子关于平时你对自己音标学习的看法，请根据你的实际情况（实际感受）作答。答案没有对错之分，对每一个句子无须多考虑。

完全不正确 有点正确 多数正确 完全正确

1. 如果我尽力去学习英语音标的话，我总是能够解决问题的。
2. 即使别人都觉得学习音标很困难，我仍然有能力进行音标学习。
3. 对我来说，坚持学好音标的理想和达成音标学习目标是轻而易举的。
4. 我自信能有效地应付任何音标学习的事情。
5. 以我的才智，我定能应付音标学习中意料之外的情况。
6. 如果我付出必要的努力，我一定能解决大多数音标学习的难题。
7. 我能冷静地面对音标学习的困难，因为我信赖自己处理音标学习问题的能力。
8. 面对一个音标学习的难题时，我通常能找到几个解决方法。
9. 有音标学习的麻烦的时候，我通常能想到一些应付的方法。
10. 无论什么音标学习的有关的事在我身上发生，我都能应付自如。

第三部分 学习音标动机量表

下面问题是关于音标学习过程中的一些做法，请根据自己的实际情况，选择对应的选项

A、非常同意 B、同意 C、一般 D、不同意 E、非常不同意

1.很多英语成绩好的同学都是用音标记单词的，我学习音标是向他们学习
2. 我学习音标是因为音标是学好英语的前提和基础。
3. 我学习音标是因为有很多人推荐学。
4. 我学习音标是为了将来成为老师后的英语教学。
5.我学习音标的动力很大程度上取决于是否喜欢英语老师。
6. 我学习音标是为了掌握英语发音基础。
7. 我学习音标是因为想纠正自己的发音。
8. 我学习音标的动力很大程度上取决于是否喜欢我的英语班级。
9. 我学音标是因为这是课本本身的学习内容。
10. 我学习音标是希望可以提高听力或口语成绩。
11. 学好音标对我很重要，因为它能提高我和别人用英语交流沟通的效率。
12. 学好音标能让我获得成就感。

13. 我学习音标，是为了更好地学习英语。

14. 我对音标学习有特别的爱好。

15. 我学习音标是为了方便自主学习新单词。

16. 我觉得音标学习十分有趣。

17. 英语是人生前进路上一块重要的敲门砖，因此学好音标很重要。

18. 我学习音标是为了可以拼读单词。

19. 我学习音标是因为我喜欢音标本身。

20. 我学习音标是为了提高语感。

21 我学习音标是因为老师强调了音标的重要性

第四部分 学习策略量表

说明：该问卷用来调查你的语言学习策略使用情况，以下答案无对错好坏之分，请根据 你的实际情况选择。谢谢你的支持与合作！1代表“从来不这样做”，2代表“几乎不这样做”，3代表“有时这样做”，4代表“经 常这样做”，5代表“总是这样做”选择符合你的实际情况的数字，并将它圈起来。

序号	内容	从来不	几乎不	有时	经常	总是
1	学英语时我会把新学的知识与已学的联系起来，建立关系网	1	2	3	4	5
2	我把新学的单词用在句子中，以加深记忆	1	2	3	4	5
3	我尽量将单词的音、形、义结合起来记忆单词	1	2	3	4	5
4	我通过在脑中联想单词的适用情况以便于记忆该单词	1	2	3	4	5
5	我利用单词的音律来记忆单词（如：rice 和 ice）	1	2	3	4	5
6	我利用生词卡片背诵新单词	1	2	3	4	5
7	我借助肢体语言记忆新单词	1	2	3	4	5
8	我经常复习学过的单词或课文	1	2	3	4	5
9	我通过记住单词在某页码或单词表中出现的位置来记忆该单词	1	2	3	4	5
10	我重复多次地读或者写新单词	1	2	3	4	5
11	我模仿以英语为母语的人的说话方式	1	2	3	4	5
12	我练习英语发音	1	2	3	4	5
13	我通过各种方式练习已经掌握的英语单词。	1	2	3	4	5
14	我常对自己说英语或者用英语与同学、老师对话	1	2	3	4	5
15	我看英语节目、英语电影或收听英语广播	1	2	3	4	5

16	我把英语阅读当做一种乐趣	1	2	3	4	5
17	我用英语记笔记、写信、写留言或者写报告	1	2	3	4	5
18	阅读文章时，我先快速浏览一遍，再重新仔细阅读	1	2	3	4	5
19	我学新单词时总是想找意义与之对应或接近的汉语词汇	1	2	3	4	5
20	我尽力总结英语中的一些固定模式比如动词搭配等	1	2	3	4	5
21	我把新单词（尤其是多音节的）分解成有意义的小部（比如前、	1	2	3	4	5
	中、后缀或同根词等等）以求明白整个单词的意思					
22	阅读中，我尽量不把英语逐字地翻译成汉语	1	2	3	4	5
23	我会总结听到或读到的英语知识	1	2	3	4	5
24	对于不熟悉的单词，我就猜测它们的意思	1	2	3	4	5
25	在英语对话中想不起表达我意思的单词时，我借助手势表达	1	2	3	4	5
26	当我不知道该用哪个单词来表达我的意思时，我会杜撰个单词	1	2	3	4	5
27	在阅读英语文章时，我不会去查每一个生词的意思	1	2	3	4	5
28	与他人进行英语对话时，我会猜测对方接下来要说什么	1	2	3	4	5
29	如果想不起恰当表达意义的单词，我会使用相似意义的单词或词 组	1	2	3	4	5
30	我尽力把新学到的单词有意识的运用到各种英语表达之中	1	2	3	4	5
31	我会留意在使用英语过程中的错误，修改错误可以帮助我更好地 学习英语	1	2	3	4	5
32	当别人说英语时，我总注意听	1	2	3	4	5
33	我一直摸索如何有效地学习英语	1	2	3	4	5
34	我计划好每天或者每阶段足够学习英语的时间	1	2	3	4	5
35	我经常找人和我一起练习口语	1	2	3	4	5
36	我尽可能多地阅读英语文章	1	2	3	4	5
37	对于提高我的英语技能，我有明确的目标	1	2	3	4	5
38	我常反思自己的英语学习方法，不断总结优缺点，不断改进	1	2	3	4	5
39	每当我对使用英语有恐惧感的时候，我试着放松自己的紧张情绪	1	2	3	4	5
40	尽管我害怕出错，但我还是鼓励自己讲英语	1	2	3	4	5
41	当我的英语表现不错时，我会用自己的方式奖励自己	1	2	3	4	5
42	我留意自己学习或使用英语时是否有紧张情绪	1	2	3	4	5
43	我把自己学习英语的感受记在日记中	1	2	3	4	5
44	我与别人交流学习英语时的体会和感受	1	2	3	4	5
45	当我不明白别人讲的英语时，我会请求说话人讲慢点或重复一遍	1	2	3	4	5
46	讲英语时，我请说英语的人更正我的英文表达	1	2	3	4	5

47	我与其他同学一起练习英语	1	2	3	4	5
48	我积极寻求英语本族者或英语老师的帮助	1	2	3	4	5
49	我主动用英语向别人提问题	1	2	3	4	5
50	我主动了解英语国家的文化	1	2	3	4	5

第五部分 学习环境量表

说明：1、各题目所述内容如与你所处现状相符，请选同意或非常同意； 如与你所处现状不 符，请选不同意或非常不同意 2、对难以做出选择的题目，请选择中立 3、问卷均为单项题， 请在您认为最合适的答案前的〇上打 √

谢谢您的参与！

项目		非常同意	同意	中立	不同意	非常不同意
1	学校有专门的英语实训场所	〇4	〇3	〇2	〇1	〇0
2	英语课上老师常用多媒体设备教学	〇4	〇3	〇2	〇1	〇0
3	校园网能够提供充足的网络链接到内部资源 （如：历年英语试卷，讲义）	〇4	〇3	〇2	〇1	〇0
4	英语上课时间安排合理	〇4	〇3	〇2	〇1	〇0
5	校园网能够提供充足的网络链接到外部资源 （如：英语相关课程网站）	〇4	〇3	〇2	〇1	〇0
6	老师使用过相关网络工具（如QQ、博客、BBS 等）布置作业及给学生答疑	〇4	〇3	〇2	〇1	〇0
		〇4	〇3	〇2	〇1	〇0
7	英语有相应的实践课程安排	〇4	〇3	〇2	〇1	〇0
8	我们的英语测试更多侧重在学生的笔试能力	〇4	〇3	〇2	〇1	〇0
9	英语听说训练绝大部分在语音教室上	〇4	〇3	〇2	〇1	〇0
10	英语老师不以权威自居， 而是像一个与我平等的朋友	〇4	〇3	〇2	〇1	〇0
11	我们经常使用网上英语材料	〇4	〇3	〇2	〇1	〇0
12	英语老师知识渊博，见识广。	〇4	〇3	〇2	〇1	〇0

13	我经常借助课外材料学习英语（参考书、英语期刊杂志等）。	○4	○3	○2	○1	○0
14	我选修过其它英语课程。					

15	该英语教材要求学生以问题解决法进行学习	○4	○3	○2	○1	○0
16	老师总是根据卷面成绩评定我们的英语水平	○4	○3	○2	○1	○0
17	我通过相关网络工具（如：QQ、博客、BBS 等）提交过作业及与老师交流	○4	○3	○2	○1	○0
18	英语老师注重学生英语学习的自我评价	○4	○3	○2	○1	○0
19	课后的作业题目设计得很好	○4	○3	○2	○1	○0
20	我课后自习的场所主要集中在教室	○4	○3	○2	○1	○0
21	我们班同学的英语学习积极性很高	○4	○3	○2	○1	○0
22	老师针对学生学习情况布置不同的作业	○4	○3	○2	○1	○0
23	我经常和同学合作一起完成英语学习任务	○4	○3	○2	○1	○0
24	在自习场所里查找英语学习资料很方便	○4	○3	○2	○1	○0
25	经常有类似新东方学习机构来校进行英语学习指导	○4	○3	○2	○1	○0
26	英语老师的信息技术（如：多媒体应用能力、计算机应用能力）水平较高	○4	○3	○2	○1	○0
27	英语老师与我们关系很融洽	○4	○3	○2	○1	○0
28	现有的英语课适量刚好合适	○4	○3	○2	○1	○0
29	英语老师经常提供材料，鼓励学生自学	○4	○3	○2	○1	○0
30	我可以随时得到英语老师的帮助	○4	○3	○2	○1	○0
31	英语课教室都配有相应的多媒体设备	○4	○3	○2	○1	○0
32	我会向他人推荐选用我们的教材	○4	○3	○2	○1	○0
33	英语老师的反馈具有鼓励性	○4	○3	○2	○1	○0
34	我提出的英语问题能得到其他同学的回应	○4	○3	○2	○1	○0
35	英语课上以老师讲授为主	○4	○3	○2	○1	○0

36	英语老师发音标准、口语流利	○4	○3	○2	○1	○0
37	我提出的英语疑问会得到网友的回应	○4	○3	○2	○1	○0
38	学校对英语学习优秀的学生给予相应的奖励	○4	○3	○2	○1	○0
39	英语老师积极引导学生参与讨论	○4	○3	○2	○1	○0
40	英语教材提供了一些有效的自主学习策略（具体的个人学习方法）	○4	○3	○2	○1	○0
41	英语老师时常设置情景组织学生角色扮演练习	○4	○3	○2	○1	○0

Appendix B

Results of four hypotheses testing

**Hypotheses**	**Decisions**
**H1.** Learning strategies can directly influence Chinese L3 English learners' motivation for PSL.	supported
**H2.** The learning environment can directly influence L3 English learners' motivation for PSL.	supported
**H3.** Self-efficacy mediates the association between learning strategy and Chinese L3 English learners' motivation for PSL.	supported
**H4.** Self-efficacy mediates the association between the learning environment and Chinese L3 English learners' motivation for PSL.	supported
